# Diagnostic factors influencing orthodontic extraction decision-making: a systematic review

**DOI:** 10.3389/froh.2026.1967358

**Published:** 2026-10-30

**Authors:** Martin Baxmann, Márton Zsoldos, Krisztina Kárpáti

**Affiliations:** Department of Orthodontics and Paediatric Dentistry, Faculty of Dentistry, University of Szeged, Szeged, Hungary

**Keywords:** arch length discrepancy, artificial intelligence, cephalometric predictors, crowding severity, machine learning, orthodontic extractions, treatment planning

## Abstract

**Introduction:**

Orthodontic extraction decision-making requires integration of multiple diagnostic characteristics, yet the factors underlying extraction vs. non-extraction treatment selection have not been comprehensively synthesized across conventional clinical research and contemporary prediction models. This systematic review aimed to identify and critically evaluate the diagnostic factors influencing orthodontic extraction decisions and determine their consistency across the available evidence.

**Methods:**

PubMed, MEDLINE, Embase, Scopus, Web of Science, and the Cochrane Library were searched from inception through July 2026 for studies evaluating diagnostic factors associated with orthodontic extraction decisions or statistical or artificial intelligence-based prediction models. Two reviewers independently performed study selection, data extraction, and risk-of-bias assessment. Substantial clinical and methodological heterogeneity precluded meta-analysis, and findings were synthesized narratively.

**Results:**

Nineteen studies met the inclusion criteria. Dental crowding and tooth size -arch length discrepancy were the most consistently evaluated diagnostic factors but were typically considered alongside facial profile, incisor position, skeletal relationships, and occlusal characteristics. No consistent numerical threshold for recommending extraction was identified. Artificial intelligence-based prediction models demonstrated promising performance, with interpretable predictors substantially overlapping with diagnostic variables identified in conventional clinical studies. Methodological quality ranged from moderate to critical risk of bias, with most prediction-model studies judged to be at high risk of bias.

**Discussion:**

These findings indicate that orthodontic extraction decision-making is fundamentally multifactorial rather than governed by a single diagnostic measurement or threshold. Current artificial intelligence models should be considered decision-support tools pending higher-quality prospective research and external validation; whether image-derived or latent features provide genuinely novel determinants of extraction need remains uncertain.

**Systematic Review Registration:**

https://osf.io/x86zy

## 1 Introduction

Orthodontic treatment is used to improve the alignment and relationship of the teeth and jaws, with the broader goals of achieving functional occlusion, supporting oral health, and improving dentofacial appearance (1). One of the most consequential decisions in orthodontic treatment planning is whether permanent teeth should be removed to create space for alignment or to correct dental and facial discrepancies (1, 2). Extraction practices have changed considerably over time, reflecting shifts in treatment philosophy, diagnostic priorities, and available treatment techniques (1, 3, 4). Although contemporary extraction rates are lower than those reported during some earlier periods, extraction remains an established treatment option for selected patients, particularly when crowding, dental protrusion, or an unfavorable relationship between the upper and lower teeth cannot be adequately managed through non-extraction approaches (4).

Dental crowding occurs when insufficient space is available within the dental arch to accommodate all teeth in normal alignment (1). This space deficiency is commonly quantified as arch length discrepancy or tooth size–arch length discrepancy (TSALD), which represents the difference between the space required to align the teeth and the space available within the dental arch (1, 5). Although crowding is a major consideration in treatment planning, it is not the only factor used to determine whether teeth should be removed (2). Clinicians also consider anterior tooth position and inclination, skeletal relationships between the maxilla and mandible, occlusal relationships in the anteroposterior, transverse, and vertical planes of space, and the patient's facial profile (2, 6, 7). In a foundational analysis of clinicians' stated reasons for extraction, crowding was the most frequently cited consideration, followed by incisor protrusion, the need for profile correction, the severity of the dental relationship, and anticipated treatment stability (2). More recent studies have similarly identified lower and upper crowding, lip position, and overjet—the horizontal distance between the upper and lower front teeth—as important contributors to extraction decisions (4, 6).

Extraction decisions are therefore based on the combined interpretation of several diagnostic findings rather than a single measurement or fixed numerical threshold. Clinical examination and diagnostic records provide information on dental crowding, tooth position, facial profile, skeletal and occlusal relationships, and periodontal status, enabling clinicians to integrate multiple diagnostic findings during treatment planning (1, 2). The importance assigned to these factors may differ among clinicians, particularly in borderline cases for which extraction and non-extraction treatment are both considered feasible (2, 6, 7). Clinician-based research has shown that facial soft-tissue profile and crowding are often assigned greater importance than isolated measurements obtained from a lateral skull radiograph, commonly referred to as a cephalogram (2, 7). This variability has contributed to continued interest in identifying more objective and reproducible approaches to extraction decision-making.

Recent advances in machine learning and artificial intelligence (AI) have led to the development of new approaches for supporting orthodontic extraction decision-making. These models analyze combinations of routinely collected clinical information, including crowding, arch length discrepancy, tooth inclination, skeletal and dental relationships, facial profile, radiographs, photographs, and digital scans, to predict whether extraction or non-extraction treatment is likely to be selected (8–10). Systematic reviews have reported promising diagnostic accuracy for these models but have also concluded that the certainty of the available evidence remains low and that AI should be considered a decision-support tool rather than a replacement for clinical judgment (8–10). Importantly, these reviews have focused primarily on model performance and diagnostic accuracy rather than the clinical variables underlying extraction decisions. Consequently, the relative importance of the diagnostic factors incorporated into these models has not been synthesized across the broader orthodontic literature.

Previous systematic reviews have examined orthodontic extraction decisions from two distinct perspectives. Those of conventional orthodontic treatment have focused primarily on treatment outcomes following extraction and non-extraction therapy, including changes in facial soft-tissues, dental arch dimensions, occlusal outcomes, smile esthetics, treatment duration, and post-treatment stability (11–13). More recently, systematic reviews of AI have evaluated the diagnostic accuracy and clinical performance of machine learning models designed to predict extraction decisions (8–10). However, neither body of literature has comprehensively synthesized the diagnostic factors that influence the initial decision to recommend extraction or non-extraction treatment. Consequently, the relative importance of clinical, dental, skeletal, soft-tissue, occlusal, and emerging computational predictors has not been evaluated across observational studies, clinician decision-making studies, comparative cohorts, and prediction models. Consequently, clinicians currently lack a comprehensive evidence synthesis describing which diagnostic variables have been most consistently associated with orthodontic extraction decision-making across both conventional clinical studies and contemporary prediction models. This systematic review was therefore undertaken to identify, critically appraise, and synthesize the available evidence regarding the diagnostic factors influencing orthodontic extraction decision-making and to address the following review question: Which diagnostic factors influence orthodontic extraction decision-making, and how consistently have these factors been associated with extraction vs. non-extraction treatment planning?

## 2 Materials and methods

### 2.1 Study design and protocol registration

This systematic review was conducted in accordance with the Preferred Reporting Items for Systematic Reviews and Meta-Analyses (PRISMA 2020) statement. The review was designed to identify, critically appraise, and synthesize evidence evaluating the diagnostic factors that influence orthodontic extraction decisions, with particular emphasis on crowding severity, arch length discrepancy, cephalometric predictors, skeletal and dental characteristics, facial profile, and emerging prediction models used to distinguish extraction from non-extraction treatment planning. The review protocol was prospectively registered with the Open Science Framework (OSF; Registration No. dxvec) before study selection commenced.

Before initiating the literature search, several protocol amendments were made to improve methodological clarity and ensure consistency between the review objectives, eligibility criteria, search strategy, planned outcomes, and data synthesis. Specifically, the review objective and title were refined to emphasize diagnostic factors influencing orthodontic extraction decision-making rather than crowding thresholds alone. The search strategy was refined through iterative pilot testing to optimize sensitivity and specificity while maintaining alignment with the review question. The final validated strategy was registered before study selection. The eligibility criteria, outcome definitions, and data extraction framework were subsequently refined to comprehensively capture diagnostic variables, including arch length discrepancy, skeletal and dental characteristics, cephalometric predictors, facial profile, clinical treatment-planning factors, and prediction models based on statistical methods or artificial intelligence. In addition, the review team was updated before the review commenced, with one author removed and another added. All amendments were completed before study identification, screening, data extraction, risk-of-bias assessment, and data synthesis; therefore, none of these modifications influenced study selection, data collection, or interpretation of the findings.

### 2.2 Eligibility criteria

Eligibility criteria were established *a priori* according to the review question and PICOS framework. Within this framework, the population comprised patients undergoing orthodontic treatment planning; the intervention/exposure comprised diagnostic factors or prediction approaches used to inform orthodontic extraction decisions; comparators included non-extraction treatment decisions or alternative extraction patterns, where applicable; the primary outcome was the extraction decision, recommendation, or classification; and eligible study designs included randomized controlled trials and analytical observational studies, including cohort, case-control, cross-sectional, and prediction-model studies.

Studies were considered eligible if they evaluated one or more diagnostic factors associated with orthodontic extraction decision-making, including crowding severity, tooth size–arch length discrepancy, space analysis, skeletal or dental characteristics, facial profile, cephalometric measurements, or other clinical predictors used to determine whether extraction or non-extraction treatment was indicated. The review focused on extractions undertaken specifically for orthodontic treatment-planning purposes, including management of crowding or arch-length deficiency, dentoalveolar compensation or orthodontic camouflage, interceptive guidance of eruption, space management, correction of dental protrusion or incisor position, and management of occlusal or dentofacial discrepancies. Eligible decisions included the initial choice between extraction and non-extraction treatment and, where applicable, selection among alternative orthodontic extraction patterns. No separate eligibility restriction was imposed according to permanent vs. deciduous tooth status. Orthognathic surgical procedures themselves were outside the scope of the review; however, studies involving surgical-orthodontic patients were not excluded solely on that basis when they evaluated diagnostic factors informing an orthodontic extraction decision.

Studies evaluating statistical prediction models, AI, machine learning algorithms, or other decision-support systems developed to assist extraction planning were also eligible for inclusion. Comparative studies examining extraction and non-extraction treatment groups were included only when baseline diagnostic variables or treatment-planning characteristics were investigated as predictors of the extraction decision rather than solely as measures of treatment effectiveness. Similarly, studies comparing different extraction patterns (e.g., two-premolar vs. four-premolar extraction) were eligible only if they evaluated the diagnostic factors or treatment-planning criteria underlying the selection of the extraction pattern, rather than reporting treatment outcomes alone.

Eligible study designs included randomized controlled trials, prospective and retrospective cohort studies, case-control studies, and analytical cross-sectional studies. Studies published in any language were eligible provided that full-text articles were available. Editorials, narrative reviews, opinion articles, conference abstracts without sufficient methodological detail, book chapters, case reports, and non-research publications were excluded. Previous systematic reviews and meta-analyses were not included in the evidence synthesis but were screened manually to identify additional eligible primary studies. Studies investigating tooth extraction for reasons unrelated to orthodontic treatment planning, such as trauma, impaction, pathology, or periodontal disease, were also excluded. Accordingly, extractions undertaken primarily for non-orthodontic therapeutic indications were outside the scope of the review.

### 2.3 Information sources and search strategy

A comprehensive electronic literature search was performed in PubMed (via MEDLINE), MEDLINE (via Ovid), Embase, Scopus, Web of Science, and the Cochrane Library from database inception through July 1, 2026. No restrictions were placed on publication year or language in order to maximize retrieval of relevant evidence. The search strategy combined controlled vocabulary, including Medical Subject Headings (MeSH) and Embase subject headings (Emtree) terms where appropriate, with free-text keywords describing orthodontics, tooth extraction, extraction vs. non-extraction treatment planning, crowding severity, arch length discrepancy, space analysis, and diagnostic predictors of extraction decisions. Search syntax was adapted to the indexing system and search functionality of each database while maintaining the same conceptual framework.

The PubMed search strategy consisted of three principal concept groups combined using Boolean operators. The first group identified orthodontic studies using the terms “Orthodontics”[Mesh], orthodont and malocclusion. The second group identified extraction-related studies using “Tooth Extraction”[Mesh] together with terms including premolar extraction, incisor extraction, serial extraction, orthodontic extraction, extraction therapy, extraction treatment, extraction protocol, nonextraction, and non-extraction. The third concept group identified studies evaluating diagnostic factors associated with extraction decisions using the terms crowding, dental crowding, tooth crowding, arch crowding, arch length discrepancy, tooth size–arch length discrepancy, TSALD, space discrepancy, space analysis, space requirements, arch perimeter, Bolton analysis, Little's irregularity index, borderline, extraction decision, extraction indication, extraction predictor, treatment predictor, extraction strategy, and extraction frequency. These concept groups were combined using the Boolean operator AND, and equivalent search strategies were translated for the remaining databases using database-specific controlled vocabulary and indexing terminology.

The PubMed search strategy was developed iteratively through pilot testing to optimize both sensitivity and specificity. Validation was performed using a predefined set of sentinel publications identified from previous reviews and expert knowledge of the orthodontic literature. The final strategy successfully retrieved all predefined reference studies addressing orthodontic extraction decision-making, crowding thresholds, extraction vs. non-extraction treatment selection, and prediction models. To minimize publication bias and maximize study retrieval, the reference lists of all included articles and relevant systematic reviews were manually screened, and forward citation searching was performed using Google Scholar, Scopus, and Web of Science when appropriate. No separate grey-literature databases or repositories were searched.

### 2.4 Study selection

All retrieved records were exported into EndNote for reference management and duplicate removal before being imported into Rayyan for study screening. Two reviewers independently screened titles and abstracts according to the predefined eligibility criteria. Articles considered potentially eligible by either reviewer proceeded to full-text assessment. Full-text screening was subsequently performed independently by the same reviewers, with reasons for exclusion documented for all ineligible studies. Disagreements at either stage were resolved through discussion, and a third reviewer was consulted whenever consensus could not be reached. The study selection process was documented using a PRISMA 2020 flow diagram.

### 2.5 Data extraction

A standardized data extraction form was developed before data collection to ensure consistency across reviewers. Two reviewers independently extracted data from each included study, and all extracted information was verified for accuracy before synthesis. Any discrepancies were resolved through discussion, with arbitration by a third reviewer when necessary. Where required information was unavailable or unclear, attempts were made to contact the corresponding study authors for clarification.

The extracted information included study characteristics (authors, publication year, country, study design, sample size, and clinical setting), participant characteristics (age, sex, malocclusion classification, skeletal classification, and baseline clinical characteristics), and the diagnostic variables evaluated for extraction decision-making. Particular emphasis was placed on crowding severity, arch length discrepancy, tooth size–arch length discrepancy, cephalometric measurements, skeletal and dental characteristics, facial profile, treatment-planning criteria, and digital or AI–based prediction models. Information on treatment allocation, reported extraction thresholds, statistical associations between diagnostic variables and extraction decisions, and principal study findings was also extracted together with any reported study limitations.

### 2.6 Outcomes

The primary outcomes of this review were diagnostic factors associated with orthodontic extraction decision-making. These included quantitative or qualitative measures of crowding severity, arch length discrepancy, skeletal and dental characteristics, cephalometric predictors, facial and soft-tissue profile, clinical treatment-planning variables, and statistical or AI–based prediction models used to support extraction decisions. Whenever reported, measures of association between these diagnostic variables and extraction treatment selection were extracted for qualitative or quantitative synthesis. The extraction-decision outcome was categorized according to how it was defined in each study, including actual treatment selection in observational cohorts, clinician-reported or case-based treatment recommendations in decision-making studies, and predicted extraction or non-extraction classification in statistical or AI-based prediction models. These outcome types were treated as distinct sources of evidence and were not assumed to represent equivalent measures of clinical decision-making.

Secondary outcomes were evaluated to provide additional clinical context regarding extraction decision-making and included extraction frequency across patient populations, post-treatment stability, relapse, changes in facial profile, occlusal and functional outcomes, long-term treatment outcomes, and temporal changes in diagnostic philosophies or extraction criteria. These outcomes were interpreted only in relation to the diagnostic factors that informed treatment planning and were not used to determine the comparative effectiveness of extraction vs. non-extraction treatment.

### 2.7 Risk of bias assessment

The methodological quality of included studies was independently evaluated by two reviewers using validated critical appraisal instruments appropriate to each study design. Randomized controlled trials were assessed using the Cochrane Risk of Bias 2 (RoB 2) tool. Observational studies were evaluated using the appropriate Joanna Briggs Institute (JBI) Critical Appraisal Checklist according to study design, including the checklists for cohort, case-control, and analytical cross-sectional studies. Studies describing statistical prediction models, machine-learning algorithms, or AI–based decision-support systems were additionally assessed using the Prediction Model Risk of Bias Assessment Tool (PROBAST) to evaluate potential bias related to participant selection, predictor assessment, outcome definition, and analytical methodology. Any disagreements between reviewers were resolved through discussion or consultation with a third reviewer. The results of the quality assessments were summarized using tabular and graphical presentations and were considered during interpretation of the review findings.

### 2.8 Data synthesis

Study characteristics and findings were synthesized using structured evidence tables and a narrative synthesis conducted in accordance with the Synthesis Without Meta-analysis (SWiM) reporting guideline. Following completion of study selection and data extraction, substantial clinical and methodological heterogeneity was identified across the included studies. Clinical and methodological heterogeneity was evident in study design, patient populations, malocclusion classifications, diagnostic variables evaluated, measurement methods, statistical approaches, prediction algorithms, and reported outcome metrics. Consequently, quantitative meta-analysis was not considered appropriate.

Studies were grouped according to the principal diagnostic domain investigated, including crowding severity and arch length discrepancy, cephalometric and skeletal characteristics, facial profile and soft-tissue considerations, clinical treatment-planning variables, and statistical or AI-based prediction models. Because definitions and measurement methods varied across studies, conceptually related diagnostic variables were grouped within these broader domains for narrative comparison while retaining the definitions, measurement methods, and units reported by the original studies. No numerical conversion or standardization was performed across non-equivalent measures. Within each domain, findings were synthesized by comparing the consistency of reported diagnostic factors, the direction of observed associations with extraction decisions, and the relative importance assigned to each predictor. Consistency was judged according to the frequency with which individual diagnostic variables were identified across studies, the agreement in the direction and interpretation of their reported associations, and the extent to which similar findings were observed across different study designs and patient populations. Greater emphasis was placed on adjusted analyses, multivariable prediction models, and studies judged to be at lower risk of bias. Methodological quality was considered during interpretation of the evidence, and differences in study design, participant characteristics, and analytical methods were incorporated into the overall assessment of the findings.

### 2.9 Certainty of evidence

Because quantitative meta-analysis was not feasible owing to substantial clinical and methodological heterogeneity among the included studies, a formal GRADE assessment was considered inappropriate. Although GRADE may be applied to narrative syntheses in selected circumstances, the included studies evaluated diverse diagnostic variables, prediction models, patient populations, and outcome measures rather than a common intervention or clinical outcome suitable for certainty rating. Consequently, the strength of the evidence was interpreted narratively by considering methodological quality, risk of bias, consistency of findings across studies, directness of the evidence, precision where applicable, and clinical applicability.

## 3 Results

### 3.1 Study selection

The literature search identified 834 records across the electronic databases. After removal of 156 duplicate records, 678 unique records remained for title and abstract screening. Following this initial screening, 588 records were excluded because they did not meet the predefined eligibility criteria, leaving 90 articles for full-text assessment. All full-text articles were successfully retrieved and evaluated for eligibility. Of the 90 full-text articles assessed, 71 studies were excluded. Reasons for exclusion included duplicate publication (*n* = 1), not primary research (*n* = 2), outcomes not relevant to the review question (*n* = 19), study focus not aligned with diagnostic factors influencing orthodontic extraction decision-making (*n* = 33), and ineligible study design (*n* = 16) (Supplementary Table S1). Consequently, 19 studies met all eligibility criteria and were included in the qualitative synthesis. The study selection process is summarized in Figure 1.

**Figure 1:**
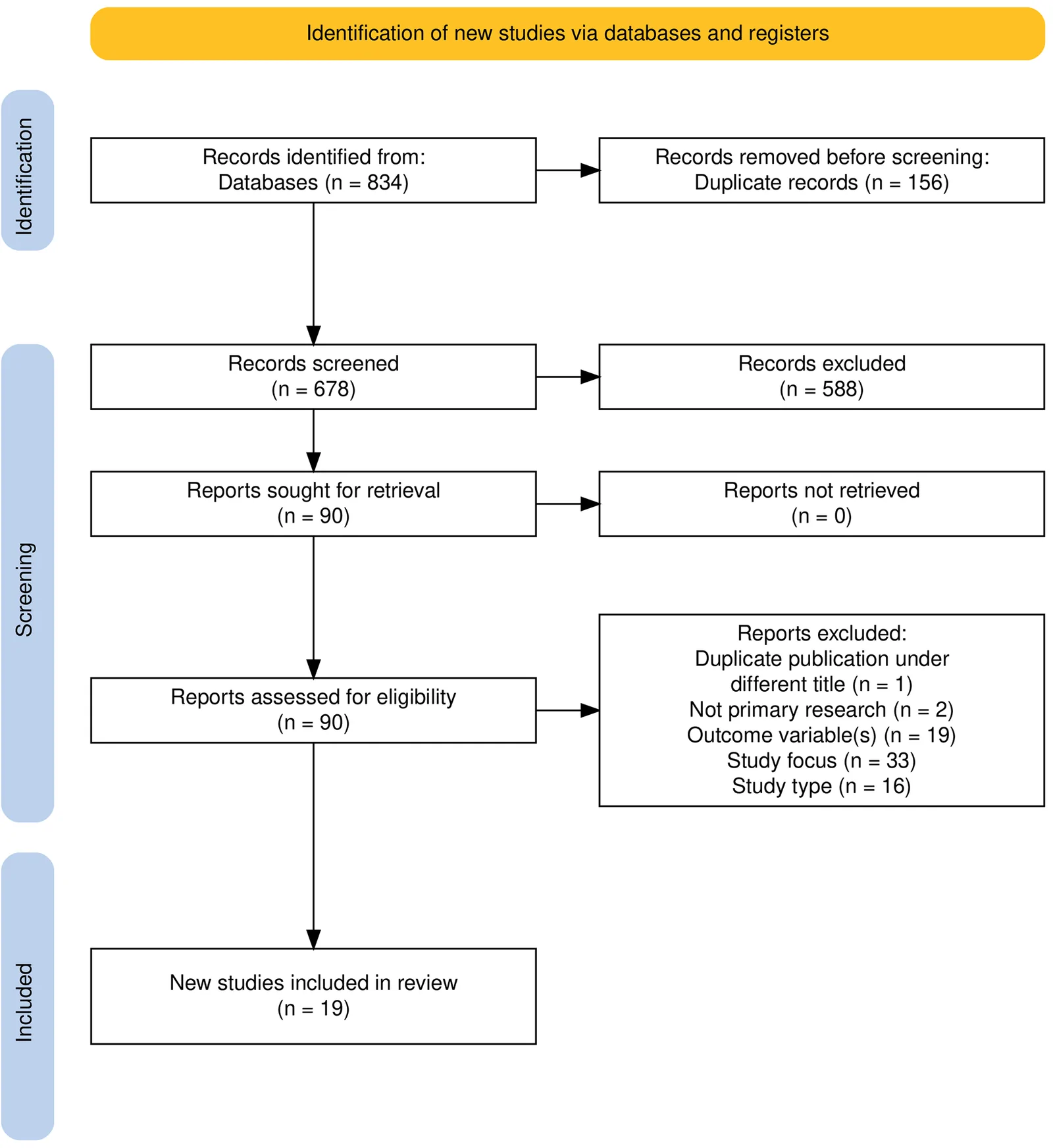
PRISMA flow diagram.

### 3.2 Study characteristics

The review included 19 studies published between 2013 and 2026, conducted across North America, Europe, Asia, and the Middle East, including the United States, China, Türkiye, Greece, Belgium, Germany, Italy, India, Iran, and South Korea. Collectively, the studies represented a broad range of orthodontic patient populations and sample sizes. Sample sizes varied substantially, ranging from 44 participants in a statistically defined borderline Class I cohort to 1,636 patients included in a deep-learning image analysis study. Most studies evaluated adolescent or young adult patients undergoing comprehensive orthodontic treatment, although several studies also included adult populations or mixed-age cohorts. The principal characteristics of all included studies are summarized in Table 1.

**Table 1:** Characteristics of the included studies.

**Author (Year)**	**Country**	**Study Design**	**Population/Sample**	**Diagnostic Variables Evaluated**	**Principal Findings**
Ahmad et al. (14)	USA/Brazil	Retrospective AI prediction model	617 orthodontic patients	Intraoral scans, cephalometric landmarks	Combined intraoral scan and cephalometric features improved extraction prediction.
Burashed (30)	USA	Retrospective cohort	121 Class I patients	Crowding, incisor position, vertical skeletal pattern	Crowding and incisor position influenced extraction pattern more than vertical dimension.
Etemad et al. (15)	USA	Retrospective machine-learning study	1,135 orthodontic patients	Crowding, cephalometric variables, overjet, overbite	Crowding was the strongest predictor of extraction decisions across institutions.
Evrard et al. (7)	Belgium	Analytical cross-sectional study	28 orthodontists evaluating 14 Class I cases	Facial profile, crowding, incisor inclination	Facial profile and crowding were the most influential clinician-reported factors.
Guo et al. (22)	China	Retrospective observational study	110 Class II Division 1 patients	Crowding, molar relationship, overjet, facial growth pattern	Lower anterior crowding was the strongest determinant of extraction strategy.
Huang et al. (16)	China	Retrospective machine-learning study	192 orthodontic patients	Crowding, facial profile, cephalometrics	Crowding, facial profile, and lower incisor inclination produced the best prediction model.
Köktürk et al. (17)	Türkiye	Retrospective machine-learning study	1,000 orthodontic patients	Arch length discrepancy, Wits appraisal, cephalometric variables	Arch length discrepancy was the strongest predictor of extraction decisions.
Konstantonis et al. (6)	Greece	Retrospective discriminant analysis	542 Class I patients	Crowding, lower lip position, overjet	Lower crowding, facial profile, upper crowding, and overjet independently predicted extraction treatment.
Leavitt et al. (25)	USA	Retrospective machine-learning study	366 extraction patients	Tooth size–arch length discrepancy, molar relationship, cephalometrics	Molar relationship and mandibular crowding were the principal determinants of extraction pattern.
Li et al. (18)	China	Retrospective neural network study	302 orthodontic patients	Crowding, cephalometrics, facial profile	Crowding was the dominant predictor of extraction decisions; sagittal discrepancy influenced extraction pattern selection.
Longerich et al. (26)	Germany	Retrospective comparative cohort	140 patients with moderate-severe crowding	Little's Irregularity Index, posterior space, skeletal morphology	Ramus Anterior Vertical measurements may assist extraction planning in borderline cases.
Mason et al. (19)	USA	Retrospective machine-learning study	393 orthodontic patients	Crowding, incisor position, cephalometrics, facial profile	Maxillary crowding was the most consistent predictor of extraction decisions.
Patrick et al. (27)	USA	Retrospective comparative cohort	346 patients (535 arches)	Crowding, second molar position, posterior space	Pretreatment second molar position and posterior space may influence extraction decisions in borderline crowding.
Ryu et al. (20)	South Korea	Retrospective AI prediction model	1,636 patients	Arch length discrepancy, crowding	Deep learning accurately predicted extraction decisions from intraoral photographs.
Saghafi et al. (28)	USA	Analytical cross-sectional study	253 orthodontists	Crowding, facial profile, mandibular incisor inclination	Crowding, facial profile, and mandibular incisor inclination were consistently identified as the most influential diagnostic factors.
Soheilifar et al. (23)	Iran	Retrospective comparative cohort	70 borderline Class I patients	Crowding, incisor position, facial convexity	Borderline extraction decisions required integration of crowding, incisor position, and facial profile.
Tepedino et al. (29)	Italy	Retrospective comparative cohort	77 adult patients	Crowding, incisor torque, anchorage, facial profile	Soft-tissue profile and crowding remained the principal considerations in extraction planning.
Verma et al. (24)	India	Retrospective comparative cohort	100 borderline Class II Division 1 patients	Arch length discrepancy, incisor protrusion, facial profile	Extraction patients demonstrated greater initial lip protrusion and incisor proclination than non-extraction patients.
Yashwant et al. (21)	India	Retrospective cohort with discriminant analysis	150 patients (44 borderline cases analyzed)	Arch length discrepancy, mandibular incisor protrusion, cephalometrics	Maxillary and mandibular arch length discrepancy and mandibular incisor protrusion were the strongest predictors of extraction treatment.

Eight studies developed or validated statistical, machine learning, or AI-based prediction models, seven employed retrospective cohort designs comparing extraction and non-extraction treatment groups or different extraction strategies, two used analytical cross-sectional designs to investigate clinician decision-making, and one applied discriminant analysis to identify the variables that best distinguished extraction from non-extraction treatment in borderline cases. Across the included studies, the diagnostic variables evaluated encompassed crowding severity, tooth size–arch length discrepancy, cephalometric and skeletal characteristics, dental relationships, facial profile and soft-tissue measurements, treatment-planning considerations, and digital prediction models. Considerable variation was observed in study populations, malocclusion classifications, predictor definitions, measurement techniques, statistical methodologies, and reported outcomes, indicating substantial clinical and methodological heterogeneity across the available evidence.

### 3.3 Diagnostic factors influencing orthodontic extraction decisions

Crowding severity and tooth size–arch length discrepancy were the most consistently identified diagnostic factors associated with orthodontic extraction decisions, although their definitions and reported thresholds varied considerably across studies (6, 14–21). Where direction of association was evaluated, greater maxillary or mandibular crowding and greater space deficiency generally favored extraction rather than non-extraction treatment. In borderline Class I patients, maxillary and mandibular arch length discrepancy together with mandibular incisor protrusion provided the strongest discrimination between treatment groups (21), while lower crowding was the strongest discriminator in a larger Class I cohort, followed by lower lip position, upper crowding, and overjet (6). Similar patterns were observed in prediction studies, in which maxillary and mandibular crowding or arch length discrepancy repeatedly ranked among the most influential predictors of extraction selection (15–19). However, the magnitude of these associations could not be directly compared across studies because predictor definitions, statistical approaches, and reported effect measures differed substantially, and several studies reported variable rankings or classification performance rather than individual effect estimates. Reported definitions of borderline or clinically relevant crowding ranged from approximately 4–10 mm of space deficiency, with no single threshold consistently distinguishing extraction from non-extraction treatment across populations (16, 19, 23, 25–29). Collectively, the direction and consistency of the evidence support increasing crowding or space deficiency as an important contributor to extraction selection, but not as an independent or universally applicable indication for extraction. A structured synthesis of the direction, consistency, magnitude and precision, and clinical interpretation of the evidence across diagnostic domains is presented in Table 2.

**Table 2:** Synthesis of diagnostic factors influencing orthodontic extraction decisions.

Diagnostic domain	Principal factors evaluated	Direction of association with extraction	Consistency of evidence	Magnitude and precision	Clinical interpretation
Crowding and arch length discrepancy	Maxillary and mandibular crowding; arch length discrepancy (ALD); tooth size–arch length discrepancy (TSALD)	Greater crowding or space deficiency generally favored extraction (6, 15–19, 21, 23, 25–30).	Consistent across conventional clinical and prediction studies	Direct quantitative comparison was limited by heterogeneous definitions, measurements, and statistical reporting. Borderline or clinically relevant crowding ranges varied across studies, commonly spanning approximately 4–10 mm of space deficiency (16, 23, 25–29).	Crowding and space deficiency were central contributors to extraction decision-making, but no universal numerical threshold was identified.
Facial profile and soft tissue	Lower lip position; lip protrusion; facial profile; facial convexity; other soft-tissue relationships	Greater dental or soft-tissue protrusion and profile characteristics judged less favorable for the treatment objectives were generally associated with extraction when considered with other diagnostic findings (6, 7, 23, 24, 28, 29).	Generally consistent	Comparable adjusted effect estimates and confidence intervals were infrequently reported, limiting assessment of the independent magnitude and precision of individual soft-tissue predictors.	Facial and soft-tissue characteristics were particularly relevant in borderline cases and were generally interpreted alongside crowding and incisor position rather than as independent extraction criteria (6, 7, 23, 24, 28, 29).
Incisor position and inclination	U1–NA; U1–SN; L1–NB; L1–MP; related measures of incisor prominence	Greater incisor protrusion or proclination generally favored extraction when additional space was required for retraction or repositioning (6, 16, 18, 19, 21, 23, 24, 30).	Generally consistent	Measurements, predictor definitions, and statistical reporting differed across studies, preventing direct quantitative comparison of individual incisor variables.	Incisor position contributed to assessment of space requirements and desired final tooth position but was not supported as an isolated extraction criterion.
Skeletal characteristics	ANB; Wits appraisal; mandibular plane angles; anterior and posterior facial height; facial-height ratios	Sagittal skeletal measures contributed to extraction and extraction-pattern selection in several studies, whereas associations involving vertical skeletal morphology were less consistent (15, 17–19, 22, 30).	Consistent as a complementary diagnostic domain; mixed for individual vertical measures	Effect estimates were heterogeneous and generally not directly comparable. Vertical dimensions contributed to some prediction models, whereas one Class I cohort found no significant differences in vertical skeletal dimensions among extraction-pattern and non-extraction groups (17, 19, 30).	Skeletal measurements generally complemented dental and soft-tissue findings rather than independently determining extraction.
Occlusal characteristics	Overjet; overbite; molar relationship; Curve of Spee; lip incompetence	Greater overjet and particular molar relationships contributed to extraction or extraction-pattern selection, whereas other associations varied according to malocclusion and the type of treatment decision evaluated (6, 18, 22, 25).	Context dependent	Direct comparison was limited because studies evaluated different malocclusions, outcomes, and extraction patterns.	Occlusal characteristics appeared particularly relevant to selection among alternative extraction strategies and were interpreted in combination with crowding and sagittal relationships (18, 22, 25).
Posterior-space and second-molar factors	Posterior mandibular space; second-molar angulation and vertical position; available eruption space	Reduced posterior space and unfavorable second-molar position were associated with considerations relevant to extraction planning in borderline cases (26, 27).	Limited evidence	These factors were evaluated in relatively few studies and using different outcomes, preventing quantitative comparison (26, 27).	Posterior-space and second-molar characteristics may provide additional information when extraction and non-extraction are otherwise both feasible, but they are not established as general extraction criteria (26, 27).
Statistical, machine-learning, and AI prediction	Multivariable combinations of dental, cephalometric, skeletal, soft-tissue, occlusal, photographic, and intraoral-scan features	Models predominantly prioritized crowding or ALD, facial profile, incisor position, and skeletal relationships, substantially overlapping with factors identified in conventional clinical studies (14–20).	Generally consistent with conventional evidence	Reported binary extraction-classification performance varied across models; direct comparison was limited by differences in sample composition, class balance, predictor sets, validation procedures, and outcome definitions (14–20).	Current prediction models support the multifactorial nature of extraction decision-making, but their clinical applicability remains constrained by methodological heterogeneity and limited independent external validation (14–20).

AI, artificial intelligence; ALD, arch length discrepancy; TSALD, tooth size–arch length discrepancy; U1–NA, maxillary incisor to NA; U1–SN, maxillary incisor to sella–nasion plane; L1–NB, mandibular incisor to NB; L1–MP, mandibular incisor to mandibular plane.

Facial profile and soft-tissue characteristics showed a similarly consistent, although less uniformly quantified, association with extraction decision-making. In clinician-based assessments of borderline cases, facial profile and crowding were repeatedly ranked among the most influential treatment-planning considerations (7, 28). Lower lip position relative to the E-plane independently contributed to discrimination between extraction and non-extraction treatment in Class I patients (6), while greater lip protrusion and incisor proclination characterized extraction patients in borderline Class II Division 1 samples (24). Facial convexity, lip position, and incisor prominence were also considered alongside crowding in borderline skeletal Class I patients and adult extraction-planning cohorts (23, 29). These findings were directionally consistent in indicating that greater dental and soft-tissue protrusion tended to favor extraction when interpreted in conjunction with available space and the desired incisor position. Nevertheless, most studies did not report directly comparable adjusted effect estimates or confidence intervals for individual soft-tissue variables, limiting conclusions regarding their independent magnitude or precision.

Cephalometric and skeletal characteristics contributed to extraction decisions primarily as complementary rather than isolated predictors. Incisor position and inclination, including U1–NA, U1–SN, L1–NB, and L1–MP, were repeatedly evaluated across conventional and prediction studies (15, 16, 18, 19, 21, 23, 30), with greater incisor protrusion or proclination generally associated with extraction selection when additional space was required for retraction. Sagittal skeletal measures, particularly ANB and Wits appraisal, contributed to several multivariable and machine-learning models (15, 17, 18), while vertical measurements showed less consistent associations. Vertical skeletal dimensions did not significantly differ among extraction-pattern and non-extraction groups in one Class I cohort with at least 5 mm of crowding (30), whereas vertical facial dimensions contributed to prediction in other multivariable models (17, 19). Thus, the evidence for sagittal and incisor-position variables was more consistent than that for vertical skeletal morphology, and no isolated cephalometric measurement demonstrated sufficient consistency across studies to function as a stand-alone extraction criterion.

Occlusal and other treatment-planning characteristics showed more context-dependent associations. Greater overjet contributed to extraction vs. non-extraction discrimination in Class I patients and to extraction-strategy selection in Class II Division 1 patients (6, 22), while molar relationship was particularly relevant when choosing among alternative premolar-extraction patterns (22, 25). Overbite, lip incompetence, and sagittal discrepancy contributed more strongly to extraction-pattern selection than to the initial binary extraction decision in an artificial neural-network model (18). Posterior space and second-molar position may also influence treatment planning in borderline cases: among patients with 5–9 mm of crowding, second-molar eruption disturbances were less frequent in extraction than non-extraction arches, while pretreatment second-molar angulation, vertical position, and available posterior space were associated with eruption disturbances (27). Posterior mandibular space assessed using the Ramus Anterior Vertical reference line was proposed as an additional planning variable in patients with 4–9 mm of mandibular crowding, although its clinical decision value remains preliminary (26). Overall, these findings indicate that occlusal and posterior-space variables may modify extraction planning in specific malocclusions or borderline cases rather than operate as general predictors across all patients.

Statistical, machine-learning, and AI-based prediction studies largely reinforced the same diagnostic domains identified in conventional clinical evidence. Maxillary and mandibular crowding were the two highest-ranking predictors in a multi-institutional random-forest model (15), while maxillary and mandibular arch length discrepancy ranked highest in an ensemble model, followed by Wits appraisal and anterior facial height (17). Other models similarly prioritized crowding, facial profile, incisor position, and skeletal relationships (16, 18, 19). Reported discrimination varied by model and population: extraction-classification accuracy ranged from approximately 77% in a multimodal intraoral-scan and cephalometric model (14) to 94% in an artificial neural-network model (18), while models predicting specific extraction patterns generally performed less consistently than binary extraction classifiers (18, 25). Direct comparison of model performance was limited by differences in sample composition, class balance, predictor sets, validation procedures, and outcome definitions. Importantly, most models lacked independent external validation, and several predicted historical clinician decisions or expert-assigned treatment labels rather than independently established treatment necessity. Thus, although prediction performance was frequently promising, the explicitly reported influential variables substantially overlapped with those identified in conventional clinical studies, and the available evidence does not establish that current models provide novel, independently validated determinants of extraction need.

### 3.4 Risk of bias assessment

Overall, the methodological quality of the included studies ranged from moderate to critical risk of bias. Of the eight prediction model studies, seven were judged to be at high risk of bias, while one was rated as moderate. The two clinician decision-making studies were both assessed as having moderate risk of bias. Among the retrospective cohort studies, two were judged to have moderate risk of bias, three were considered to have serious risk of bias, and one was assessed as having critical risk of bias. Common methodological concerns included retrospective study designs, limited external validation of prediction models, selection bias, potential confounding due to non-randomized treatment allocation, and variability in clinician treatment philosophies. A summary of the risk-of-bias assessments for all included studies is presented in Figure 2.

**Figure 2:**
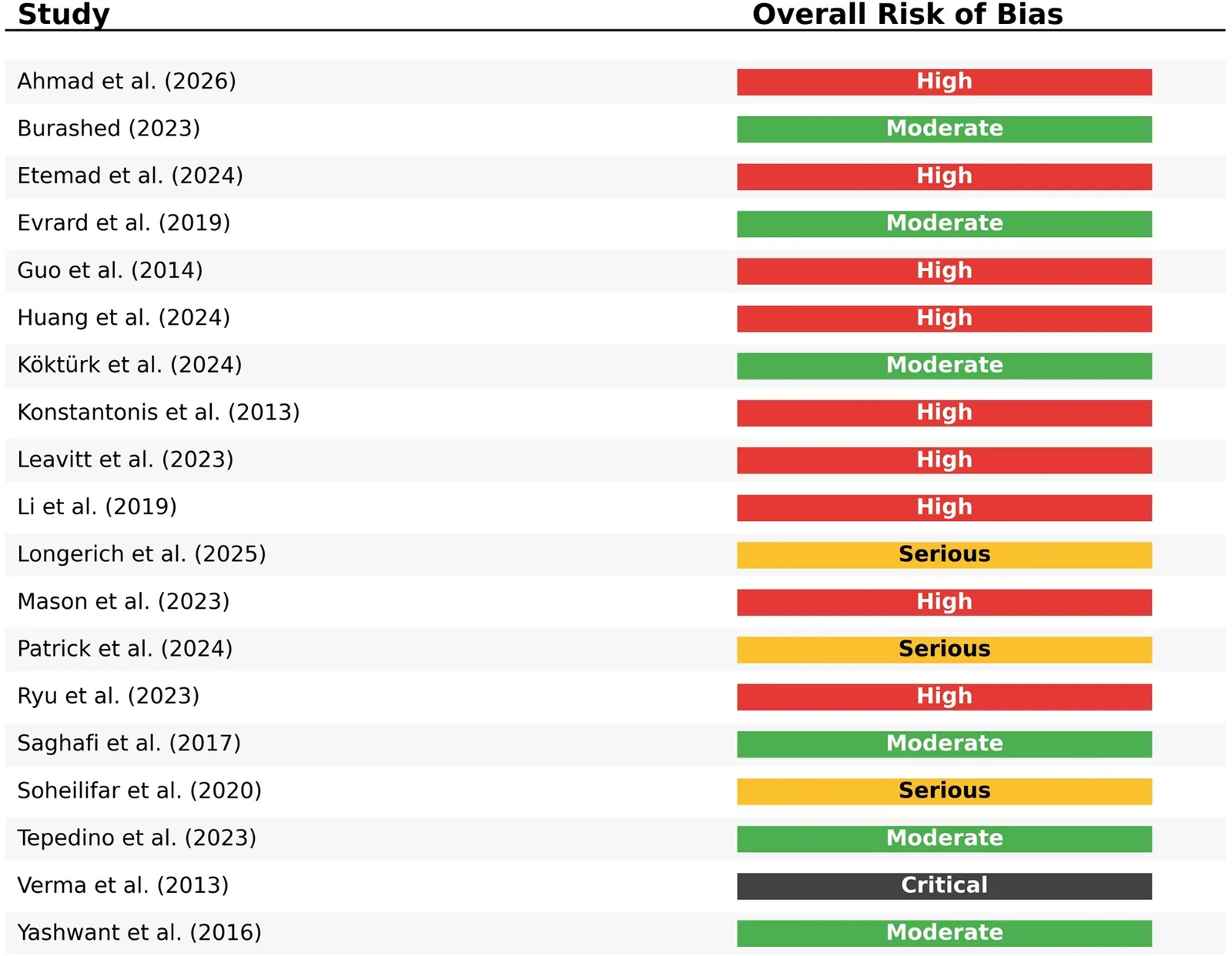
Overall risk of bias assessments.

## 4 Discussion

The available evidence suggests that orthodontic extraction decision-making is a multifactorial process in which multiple diagnostic characteristics are considered collectively rather than based on any single clinical variable. Dental crowding and tooth size–arch length discrepancy emerged as the most consistently evaluated diagnostic factors; however, they were routinely interpreted alongside facial profile, incisor position, skeletal relationships, occlusal characteristics, and other patient-specific considerations when determining whether extraction treatment was appropriate (2, 6, 7, 28). No universally accepted numerical threshold for recommending extraction was identified across the included studies, with reported crowding classifications and diagnostic thresholds varying considerably among patient populations and study designs (15, 17, 19, 21, 22, 26, 27). Although these findings consistently support the multifactorial nature of orthodontic extraction decision-making, they should be interpreted in the context of the predominantly retrospective study designs and the variable methodological quality of the included evidence. Nevertheless, the consistent identification of multiple diagnostic variables across studies suggests that extraction decisions are unlikely to be based on predefined numerical cutoff values alone (2, 4).

These findings complement, rather than duplicate, previous evidence syntheses. Earlier systematic reviews of conventional orthodontic treatment have primarily evaluated treatment outcomes following extraction and non-extraction therapy, including facial soft-tissue changes, smile esthetics, occlusal outcomes, treatment duration, and post-treatment stability (11–13). More recent systematic reviews have focused on the diagnostic accuracy of AI models developed to predict extraction decisions (8–10). In contrast, the present review synthesized the diagnostic variables underlying extraction decision-making across conventional clinical studies and prediction models, providing a broader synthesis of the diagnostic factors incorporated into orthodontic treatment planning. By synthesizing both conventional clinical evidence and prediction-model studies within a single framework, the present review provides a more comprehensive understanding of the diagnostic reasoning underlying orthodontic extraction decision-making than has previously been available.

One of the most consistent observations across the included studies was the central role of crowding and arch length discrepancy in extraction planning. Importantly, this pattern was observed not only in prediction models but also in clinician decision-making studies, where orthodontists consistently considered crowding together with facial profile, incisor position, and other patient-specific characteristics when evaluating borderline extraction cases (2, 6, 7, 28). Across these diverse study designs, crowding and arch length discrepancy consistently emerged among the most frequently evaluated diagnostic variables, reinforcing their central role in contemporary orthodontic treatment planning (6, 15, 17–19, 21, 22). However, the interpretation of crowding differed across studies, with definitions of mild, moderate, severe, and borderline crowding varying substantially and reported thresholds ranging from approximately 4 mm to more than 9 mm of space deficiency (16, 23, 25, 27, 29). This variability likely reflects differences in patient populations, diagnostic philosophies, and treatment objectives rather than disagreement regarding the importance of crowding itself (2, 4). However, because these observations were derived primarily from retrospective observational studies with heterogeneous patient populations and variable methodological quality, the relative contribution of crowding to extraction decision-making should be interpreted cautiously and within the clinical context of each study.

Facial profile and soft-tissue characteristics also remained integral components of extraction decision-making. Clinician-based studies consistently ranked facial profile among the most influential diagnostic considerations, particularly in borderline malocclusions in which both extraction and non-extraction treatment were considered feasible (7, 28). Similarly, observational studies identified lower lip position, facial convexity, lip protrusion, and mandibular incisor position as variables that contributed to treatment planning when interpreted alongside crowding and skeletal relationships (6, 21, 23, 24). These findings are consistent with the evolution of orthodontic treatment planning toward greater emphasis on facial esthetics while maintaining consideration of dental and skeletal relationships (2, 4).

Although the included AI studies consistently identified similar diagnostic predictors, their methodological characteristics varied substantially. Importantly, the prediction target in these studies generally reflected clinician-selected or expert-assigned extraction vs. non-extraction treatment decisions; therefore, reported classification performance primarily indicates agreement with these reference decisions rather than independent determination of whether extraction was clinically necessary. Sample sizes ranged from 192 to 1,636 patients, and several datasets were imbalanced with respect to extraction and non-extraction cases (14–20). Predictor selection also differed considerably, ranging from predefined clinical and cephalometric variables to intraoral-scan features, image-derived representations, and multimodal combinations of clinical and imaging data (14–20). Internal validation approaches included cross-validation and training-validation-test splits, but independent external validation was generally absent, limiting assessment of model transportability across populations and clinical settings. Reported discrimination was frequently high, with binary extraction-classification accuracies ranging from approximately 77% to 94% in the included models; however, performance metrics, class distributions, and validation procedures differed sufficiently to preclude direct comparison (14–20). Calibration and handling of missing data were inconsistently reported, while class imbalance and relatively small or single-center datasets increased the potential for overfitting in several studies. Explainability also varied: some studies quantified feature importance or used approaches such as SHAP or image-based activation mapping, whereas others provided more limited insight into how predictions were generated (14–20). These methodological limitations are consistent with the predominantly high risk of bias identified among prediction-model studies and with broader evidence identifying limited external validation, restricted dataset diversity, and insufficient explainability as persistent barriers to the clinical translation of AI in orthodontics (31). Importantly, among models based on predefined clinical and cephalometric variables, the most influential predictors—particularly crowding, arch length discrepancy, facial profile, incisor position, and skeletal relationships—substantially overlapped with variables identified in conventional clinical studies (15–19). Image-based models differed in that they could learn features directly from intraoral photographs or imaging data rather than relying exclusively on manually selected clinical variables; however, the clinical meaning of these learned representations was not always directly interpretable (14, 20). Accordingly, the available evidence demonstrates substantial overlap between interpretable AI predictors and established clinical diagnostic factors but does not establish whether image-derived or latent features represent genuinely novel determinants of extraction need. Current AI models should therefore be regarded as decision-support tools rather than replacements for clinical judgment (8–10).

The broad range of clinical populations and treatment-planning scenarios represented across the included studies should be considered when interpreting the findings of this review. Participants included adolescents and adults with Class I and Class II malocclusions, as well as patients presenting with borderline and more severe crowding (6, 21–24, 26, 27, 30). In addition, the studies differed in their primary objectives, with some evaluating the initial extraction vs. non-extraction decision, others examining specific extraction patterns, and more recent investigations developing statistical or AI-based prediction models to support treatment planning (14, 15, 17–20, 25). This clinical and methodological diversity likely contributed to differences in the reported importance of individual diagnostic variables and helps explain why no universally accepted crowding threshold for recommending extraction was identified across the available evidence. Extraction decisions may also vary according to clinician experience and treatment philosophy, patient preferences and expectations, population characteristics, and clinical practice setting, several of which were inconsistently represented or reported across the included studies. Accordingly, the associations identified in this review should not be interpreted as causal relationships or universally applicable indications for extraction. Rather, clinicians consistently interpreted crowding in conjunction with facial profile, incisor position, skeletal relationships, and patient-specific treatment objectives (2, 6, 7, 28). Consequently, crowding should be viewed as one component of a comprehensive diagnostic assessment rather than an independent indication for extraction, and the findings of this review should be interpreted within the clinical context of the populations and treatment decisions evaluated rather than as universally applicable criteria for extraction decision-making.

From a clinical perspective, the available evidence suggests the broad range of diagnostic variables reported to be associated with orthodontic extraction decision-making. Although crowding and arch length discrepancy were the most consistently evaluated factors, extraction decisions were typically considered in conjunction with facial profile, incisor position, skeletal relationships, and occlusal characteristics rather than a single diagnostic measurement. These findings underscore the complexity of orthodontic treatment planning and provide a consolidated summary of the diagnostic factors currently reported across conventional clinical studies and contemporary prediction models. This synthesis may also inform future efforts to develop and evaluate clinical decision-support systems by identifying the diagnostic variables most consistently incorporated into existing models while emphasizing the need for higher-quality evidence before such tools can be more broadly implemented in clinical practice. Despite the consistency with which several diagnostic variables were identified across the included studies, confidence in these findings remains limited by the overall certainty of the available evidence. Most studies employed retrospective observational designs or retrospective prediction-model development, many were judged to be at moderate-to-high risk of bias, and considerable clinical and methodological heterogeneity was present across study populations, diagnostic criteria, predictor definitions, and analytical approaches. Consequently, the reported associations should be interpreted as reflecting consistent patterns within the current literature rather than definitive evidence regarding the relative importance of individual diagnostic variables.

The review has several strengths. This review represents the first comprehensive synthesis of the diagnostic factors influencing orthodontic extraction decision-making across observational studies, clinician decision-making studies, comparative cohorts, and prediction models. The review also integrated contemporary AI studies alongside conventional orthodontic research, providing a comprehensive overview of both traditional and emerging approaches to extraction planning. Several limitations should be considered when interpreting the findings of this review. First, the included studies demonstrated substantial methodological and clinical heterogeneity with respect to study design, patient populations, malocclusion characteristics, diagnostic variables, outcome definitions, and analytical methods. This heterogeneity precluded quantitative meta-analysis and limited direct comparisons across studies. Second, the overall methodological quality of the included evidence was variable, with most prediction model studies judged to be at high risk of bias and several comparative observational studies assessed as having serious or critical risk of bias. These judgments primarily reflected retrospective study designs, the absence of external validation for many prediction models, potential selection bias, non-randomized treatment allocation, and limited control of confounding variables. Consequently, although consistent patterns were identified across the literature, these findings should be interpreted as representing reproducible associations across studies rather than definitive evidence of the relative importance of individual diagnostic variables. The certainty with which individual diagnostic factors can be prioritized therefore remains constrained by the quality of the available evidence. Finally, variation in the definitions of crowding severity, borderline malocclusion, and diagnostic thresholds further limited opportunities for quantitative synthesis and may have contributed to differences in the reported importance of individual diagnostic variables.

Future research should prioritize prospective, multicenter studies that evaluate extraction decision-making using standardized diagnostic criteria and consistent definitions of crowding severity and tooth size–arch length discrepancy. Greater standardization in the measurement and reporting of diagnostic variables, treatment-selection criteria, and clinician decision-making processes would improve comparability across studies and facilitate future quantitative evidence syntheses. Additional research is also needed to externally validate AI models across diverse populations and clinical settings and to evaluate their performance using standardized reporting frameworks. Finally, studies incorporating patient-centered outcomes and patient preferences into extraction decision-making may provide a more comprehensive understanding of the factors influencing contemporary orthodontic treatment planning.

## Data Availability

The original contributions presented in the study are included in the article/Supplementary Material, further inquiries can be directed to the corresponding author.
