# Supplementary material for: Diagnostic factors influencing orthodontic extraction decision-making: a systematic review
Source: Front Oral Health. 2026 Oct 30;7:1967358. doi: 10.3389/froh.2026.1967358 (PMC13759847; doi:10.3389/froh.2026.1967358)
Supplement: Supplementary file 1 [file Table1.docx]

Supplementary Material

# Supplementary Table 1. Studies excluded at full-text screening stage

| **First Author (Year)** | **Reason** |
| --- | --- |
| Aidar (2016) | Study focus |
| Al-Gumaei et al. (2025) | Outcome variable(s) |
| Aljabab et al. (2021) | Outcome variable(s) |
| Aljhani & Zawawi (2012) | Study type |
| Almeida et al. (2012) | Study type |
| Al-Taai et al. (2023) | Outcome variable(s) |
| Araújo & Caldas (2019) | Study type |
| Barreto & Santos (2018) | Study type |
| Baxi et al. (2023) | Not primary research |
| Berbert et al. (2021) | Study focus |
| Beycan & Acar (2016) | Study type |
| Burnett et al. (2025) | Outcome variable(s) |
| Çakmak et al. (2026) | Outcome variable(s) |
| Changsiripun & Phusantisampan (2017) | Study type |
| Çoban Büyükbayraktar et al. (2025) | Study type |
| Cunha et al. (2017) | Study type |
| Dai et al. (2019) | Study focus |
| Dinçer et al. (2013) | Study focus |
| Dzingle et al. (2026) | Outcome variable(s) |
| Elkholy et al. (2025) | Outcome variable(s) |
| Fathalla et al. (2023) | Outcome variable(s) |
| Ferrillo et al. (2026) | Study focus |
| Gaddam et al. (2015) | Study focus |
| Gambardela-Tkacz et al. (2023) | Study focus |
| Gaur et al. (2016) | Study type |
| Ghasemi et al. (2026) | Study focus |
| Giannakopoulou et al. (2025) | Study focus |
| Grassia et al. (2020) | Study focus |
| Guirro et al. (2016) | Study focus |
| Guo et al. (2025) | Outcome variable(s) |
| Hwang et al. (2019) | Study type |
| Janson et al. (2021) | Study focus |
| Jung (2013) | Study focus |
| Kamal et al. (2016) | Study focus |
| Khalil et al. (2025) | Not primary research |
| Konstantonis (2012) | Outcome variable(s) |
| Kumar et al. (2013) | Outcome variable(s) |
| Machado (2015) | Study type |
| Mahtani & Jain (2020) | Study focus |
| Makrygiannakis et al. (2024) | Study focus |
| Mang de la Rosa et al. (2023) | Outcome variable(s) |
| Mascarenhas et al. (2015) | Study type |
| Mladenovic et al. (2024) | Outcome variable(s) |
| Mladenovic et al. (2024) | Study focus |
| Mortezai et al. (2023) | Outcome variable(s) |
| Nerurkar et al. (2023) | Study focus |
| Okazaki (2010) | Outcome variable(s) |
| Perkovic et al. (2023) | Outcome variable(s) |
| Persson et al. (2022) | Outcome variable(s) |
| Pisc et al. (2025) | Study focus |
| Ren et al. (2022) | Study focus |
| Richardson et al. (2023) | Study focus |
| Rübendiz et al. (2026) | Outcome variable(s) |
| Sayahpour et al. (2025) | Study focus |
| Serbanoiu et al. (2024) | Study focus |
| Shafique et al. (2023) | Study focus |
| Shah & Shah (2016) | Study type |
| Sonis & Ackerman (2011) | Study focus |
| Stupar et al. (2016) | Study focus |
| Tang et al. (2025) | Study focus |
| Teng et al. (2019) | Outcome variable(s) |
| Tüfekçi et al. (2025) | Study type |
| Varghese et al. (2016) | Study focus |
| Vejwarakul et al. (2023) | Study focus |
| Verma et al. (2013) | Duplicate publication under different title |
| Wong et al. (2022) | Study focus |
| Xing et al. (2023) | Study type |
| Yang et al. (2025) | Study focus |
| Zafarmand & Zafarmand (2015) | Study focus |
| Zawawi (2014) | Study type |
| Zhang et al. (2025) | Study focus |
